# Why do *Varroa* mites prefer nurse bees?

**DOI:** 10.1038/srep28228

**Published:** 2016-06-15

**Authors:** Xianbing Xie, Zachary Y. Huang, Zhijiang Zeng

**Affiliations:** 1 Department of Laboratory Animal Science, Nanchang University, Nanchang, Jiangxi, China; 2 Honeybee Research Institute, Jiangxi Agricultural University, Nanchang, Jiangxi, China; 3 Department of Entomology, Michigan State University, East Lansing, MI, USA; 4 Ecology, Evolutionary Biology and Behavior, Michigan State University, East Lansing, MI, USA

## Abstract

The *Varroa* mite, *Varroa destructor,* is an acarine ecto-parasite on *Apis mellifera.* It is the worst pest of *Apis mellifera*, yet its reproductive biology on the host is not well understood. In particular, the significance of the phoretic stage, when mites feed on adult bees for a few days, is not clear. In addition, it is not clear whether the preference of mites for nurses observed in the laboratory also happens inside real colonies. We show that *Varroa* mites prefer nurses over both newly emerged bees and forgers in a colony setting. We then determined the mechanism behind this preference. We show that this preference maximizes *Varroa* fitness, although due to the fact that each mite must find a second host (a pupa) to reproduce, the fitness benefit to the mites is not immediate but delayed. Our results suggest that the Varroa mite is a highly adapted parasite for honey bees.

The *Varroa* mite *(Varroa destructor*) is an external parasite of the Western honey bee (*Apis mellifera*), and is by far its worst pest worldwide. Nearly all scientists agree that the mite plays a large, if not the major, role in causing colony losses in both USA^1^ and around the world^2^. The mite’s highly specialized life cycle has two phases: a phoretic stage on adult bees, and a reproductive stage on pupae^3^. During the phoretic stage, mites feed on and frequently switch among adult bees, but cannot increase their population. Phoretic stage must have a physiological role for mites, because mites not only cannot increase their population, but also experience higher mortality during this stage by falling from the hosts or being groomed off by other workers^3^. However, mites without phoretic stage could reproduce up to eight cycles and the average number of offspring for the first five cycles was four^4^. However, no study has compared fitness of mites with phoretic stage to these without, to determine the contribution of nutrition, if any, obtained during phoretic stage.

*Varroa* mites show high host specificity, which requires them to select appropriate hosts at a special stage. Varroa is able to distinguish drone larvae from worker larvae^5^ and this is most likely due to different host odors^6^. *Varroa* mites clearly prefer nurses when they are presented a choice between a forager and a nurse^7^,^8^,^9^. However all these studies were conducted inside laboratories using either live or freshly frozen bees. For field studies, one study showed mite preference for 6 and 12 day old bees^10^, but it appeared to have used only one colony. Another study showed no difference for mite preference for 6–13 (nursing age) over 17–29 day old bees (foraging age), again using a single colony but with two cohorts of bees^11^. Thus mite host preference inside colonies is inconclusive. In addition, in both studies the mite preference was studied by following the same cohort of bees as they aged, so environmental conditions were also changing, both internal and external to the single colony used. In this study we tested whether mites prefer nurses over other bees when they are given simultaneously a choice of three types of bees in field colonies, and whether feeding on nurses increases their fecundity or fitness when they later reproduce on worker pupae.

## Results

### Host preference experiment

**Figure 1:**
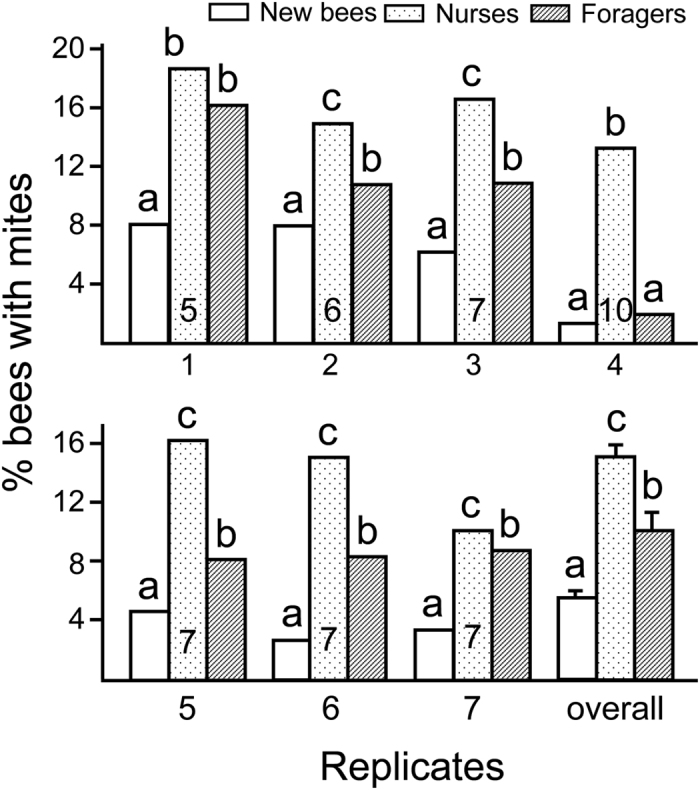
Preference by *Varroa* mites for different types of bees as phoretic hosts, shown as percentage of bees with a mite. Nurses had the highest proportion with mites, followed by foragers and then newly emerged bees (“new bees”). Results based on seven trials (each trial using a different source colony), with 1–4 in Michigan, USA, and 5–7 in Jiangxi, China. Numbers inside each “nurse” bar indicate age of the nurses. Different letters on top of each bar denote significant differences (P < 0.05) using G-tests for individual trials, or Fisher’s Protected Least Significant Difference Tests after analysis of variance showed a significant effect (Overall).

In 7 triple cohort colonies, on average we recovered 88.0 ± 3.6% (Mean ± SE) newly emerged bees (“new bees”), 88.0 ± 4.5% nurses and 80.4 ± 2.4% foragers. There were no significant differences among these recovery rates (ANOVA, F_2, 18_ = 1.44, *P* = 0.26). We assumed that each mite attached to a different host, so the mite/bee ratio in each group was used as an estimate for percentage of bees with mites, as was done in other studies^10^,^11^. Analyzing the data from each colony individually, all colonies showed significant differences among the three types of bees in mite distribution (Fig. 1), with the exception of Colony 1 (no difference between foragers and nurses) and 4 (no difference between newly emerged bees and foragers). Analyzed together, the effect of worker age on mite distribution was highly significant (ANOVA, F_2, 18_ = 23.28, *P* < 0.01), with nurses having the highest number of mites, followed by foragers, then newly emerged bees (Fig. 1).

### Mite fecundity and fitness after feeding on different phoretic hosts

**Figure 2:**
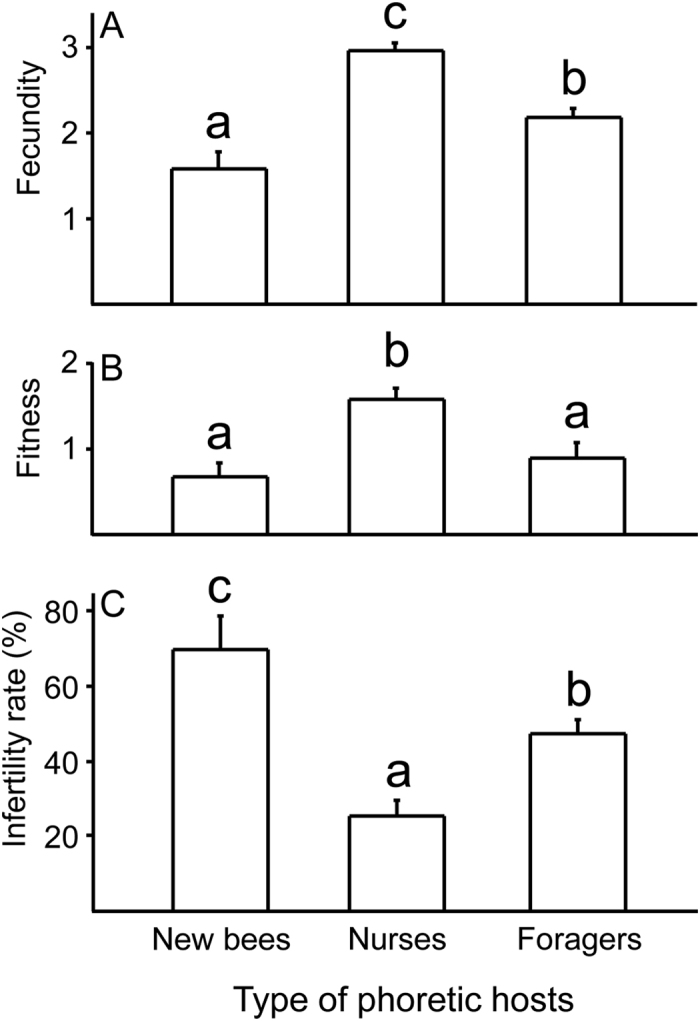
Number of total female offspring (fecundity) (**A**), number of mature daughters (fitness) (**B**) and infertility rate (**C**) after mites had fed on different types of phoretic hosts. Different letters on top of each bar denote significant differences (P < 0.05) using Fisher’s Protected Least Significant Difference Tests after analysis of variance showed a significant effect. Each bar represents data from 4 trials, each trail with N = 35 mites. The data included mites that were infertile (non-reproducing).

Mite fecundity was significantly affected (ANOVA, F_2, 9_ = 23.61, *P* < 0.01) by the type of phoretic hosts that they had previously fed upon: mites that fed on nurses showed the highest number of female offspring, followed by those fed on foragers, then these on newly emerged bees (Fig. 2A). Mite fitness followed a similar pattern, (ANOVA, F_2, 9_ = 9.15, *P* < 0.01) with mites that hosted by nurses having significantly higher fitness than the mites on the other two types of bees, but mites feeding on foragers showed the same fitness as those on newly emerged bees (Fig. 2B). Infertility rates also differed significantly among the mites hosted by the three types of hosts (ANOVA, F_2, 9_ = 14.42, *P* < 0.01), with mites fed upon nurses having the lowest infertility rate, followed by foragers, then newly emerged bees (Fig. 2C).

**Figure 3:**
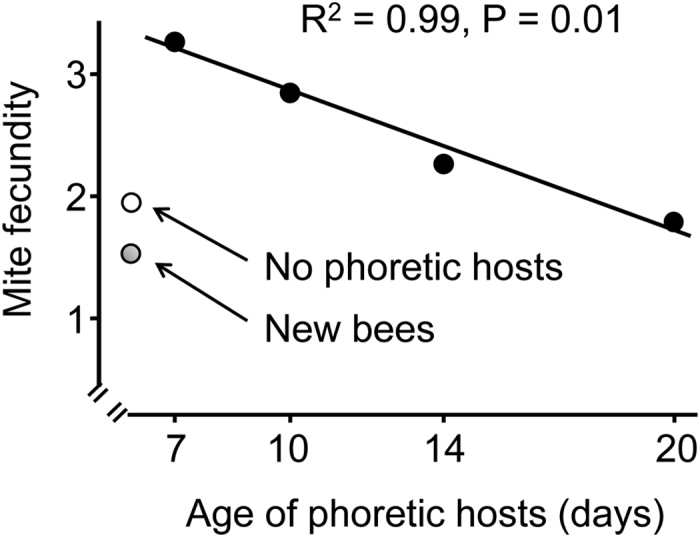
Mite fecundity (# of female offspring per mother mite) after they fed on phoretic hosts of different ages. There was a significant negative effect of age of phoretic hosts on mite fecundity based on regression analysis (R^2^ = 0.99, P = 0.01). Each point represents data from 26–35 mother mites. The data included mites that were infertile (non-reproducing). Data for no phoretic hosts were estimated from Ruijter^4^ from the first 2 reproductive cycles, after subtracting the males from total eggs laid and then factored with 80% of fertility. Data for newly merged bees were from Fig. 2A (1.58 ± 0.2).

Finally, when mites were hosted by phoretic hosts with known ages, there was a significant negative relationship between mite fecundity and age of phoretic hosts (R^2^ = 0.99, *P* = 0.01, Fig. 3).

## Discussion

Our study clearly demonstrated that 1). *Varroa* mites preferred nurses over older (foragers) and younger (new) bees, even inside a colony setting; 2). The type of phoretic hosts affected mite reproduction, with nurses resulting in the highest fitness for mite reproduction. This higher fitness is particially due to lower infertility rate in mites that had fed on nurses; and 3). There was a significant negative relationship between the age of the phoretic hosts and mite fecundity when we used only known-aged bees.

In this study, we presented to *Varroa* mites choices of newly emerged bees, nurses and foragers in a “triple cohort colony”. This type of colony has been shown previously to induce normal behavioral development in the middle-aged group (nurses)^12^. We thus assumed that the age demography would also appear to be normal to the mites. Because there were no differences in recovery among the three groups of bees, the preference of host could be described by the percentage of bees with mites in each group. Among the 7 triple cohort colonies, *Varroa* mites clearly preferred to parasitize on nurses over foragers, and foragers over newly emerged bees (Fig. 1). These results are consistent with previous laboratory studies^7^,^8^,^9^, a study using a single colony^11^, and a more recent study which also used natural colonies^13^. In that study mites were also shown to avoid foragers in field colonies but this avoidance disappeared when mite density is high. Thus *Varroa* mites not only are able to choose drones as reproductive hosts^14^, they clearly prefer nurses over younger or older bees not only in Petri dishes or caged bees, but also at more natural colony settings.

Why do phoretic mites prefer nurses? One possibility is that foragers are avoided due to higher risks when foraging (natural mortality and being preyed upon)^15^, while nurses are more appealing, since they contact larvae frequently for feeding and insepction^16^, allowing *Varroa* mites greater opportunity to enter into brood cells. Alternatively, or in addition to the aforementioned reasons, nurses may provide better nutrition as phoretic hosts, resulting in higher fecundity when mites later enter cells to reproduce. We found that the fecundity of mites that fed upon different phoretic hosts followed the same exact sequence of host preference: with mites feeding on nurses showing the highest fecundity, followed by mites that had fed on foragers, and those fed on newly emerged bees having the lowest fecundity (Fig. 2A). It is possible that mites could have a higher number of offspring but their actual fitnesses remain the same. This is because most mite offspring have a low probability to become mature when their worker hosts emerge^17^. We therefore also estimated fitness by including only mature daughters and these daughter mites that could potentially become mature when their host was ready to emerge (old deutonymphs). We found a similar pattern as mite fecundity, although now the difference between foragers and newly emerged bees disappeared (Fig. 2B). Our estimated mite fitness (1.60 ± 0.13, Fig. 2B) was similar to those reported previously in mites reproducing in natural colonies. For example, Martin^17^ estimated an average fitness to be 1.45, and Fries *et al.*^18^ found that each invading mother mite can produce about 1.5 mated female offspring in worker cells.

We let the mites feed on phoretic hosts for only 3 days while the average phoretic duration is about 6 days^3^. However, even at 3 days we found a significant difference in either fecundity or fitness. It is possible that differences will be higher, not smaller in the natural colonies when mites feed on phoretic hosts for a longer time. We did not let mites feed for 6 days because by then the newly emerged bees will become “nurses” physiologically and “foragers” will also revert to become mostly nurses after such a long time^12^.

How did nurses as phoretic hosts increase mite fecundity and fitness? They could either decrease the proportion of non-reproducing mites (infertility rate) or cause the mites to lay eggs sooner or more frequently, thus allowing more daughters to mature. Our data of reduced infertility rates in mites fed on nurses (Fig. 2C) suggests that it is the first mechanism. Causing infertility in mites can be a powerful mechanism to resist *Varroa*. For example *Apis cerana* is highly resistant to *Varroa* and mites invading worker cells show 100% infertility; the Africanized bee (*A. mellifera scutellata*) was intermediately resistant and has a 40% infertility rate; while *A. mellifera* in U.S. is the least resistant with the lowest infertility rate (10–20%) in worker brood^19^. Our data here provides the first evidence that phoretic host type can also affect infertility rate of *Varroa*.

Lastly, we found a negative relationship between the age of phoretic hosts and the fecundity of mites, with older bee hosts providing a significantly lower mite fecundity (Fig. 3). Therefore, even though there is a 15 day delay (6 days average phoretic period +12 days worker capped brood period), *Varroa* mites still correctly “planned” their future reproduction gain by selecting the most suitable phoretic hosts. While it has been shown previously that *Varroa* prefers drone pupae as reproductive hosts^14^ to result in a fitness gain, we show here for the first time that phoretic hosts also has a significant impact on mite reproduction. Mites feeding on nurses showed almost 2 times higher fitness than those feeding on newly emerged bees (Fig. 2A). We did not have a group without phoretic hosts, so we cannot conclude how poorly a nutrition newly emerged bees or foragers are providing to mites. Using 1^st^ and 2^nd^ reproductive cycles of mites that bypassed phoretic hosts^4^, we calculated the number of female offspring (total minus males, factored by 80% fertility of mites) to be 1.9, slightly lower than the fecundity of mites feeding on foragers (2.19 ± 0.10, Fig. 2A), but slightly higher than fecundity of mites fed on 20 day old workers or newly emerged bees (Fig. 3). These numbers are most likely not statistically different. This suggests that both old bees (or foragers) and newly emerged bees lacked some nutrition and these mites performed the same as mites which had no phoretic hosts.

Although *A. mellifera* is a relatively new host for *V. destructor*, the mites are nevertheless able to maximize their fitness by preferring the “correct” phoretic host. This preference most likely evolved while mites were still on their original host *Apis cerana*. It is not clear what component(s) in the nurses’ blood increase mite reproduction, but it is known that nurses are characterized with a very different physiology. These include lower juvenile hormone titers^20^, higher vitellogenin^21^ and protein^22^ titers in hemolymph, and more fat in the abdomen^23^. Future studies should focus on identifying the chemicals responsible for decreasing mite reproduction (as is the case for foragers as phoretic hosts) and utilizing them to reduce the reproduction of this serious honey bee parasite.

## Materials and Methods

### Host preference experiment

We constructed experimental colonies each with three age cohorts^12^. Each colony was consisted of 500 newly emerged bees (<24 h after emergence), 500 nurses (5–11 day old bees reared in a hosting colony) and 500 foragers (returning bees with pollen loads, of unknown ages; but in typical colonies they are older than 21 days). All source colonies were surveyed to make sure they were of low mite numbers. The nurses were bees paint-marked upon emergence, introduced into their natal colonies and then recovered when they were between 5 and 11 days old via an insect vacuum (BioQuip, USA). Newly emerged bees were obtained from the same source colony by incubating brood again. Newly emerged bees were brushed into cat-litter pans coated with vegetable oil (Pam®) so that they could be painted by a Testor’s enamel paint. Foragers were obtained by blocking the entrance of the same colony using hardware clothe and vacuuming the returning bees with pollen on their corbiculae^12^. Each “triple cohort colony” had a queen, a frame with 1–3 day old larvae, and a frame with pollen and honey as food. Colonies were placed on a stand in the field and allowed to forage freely. *Varroa* mites (N = 200 per colony) were harvested from workers using powdered sugar^25^, cleaned of the sugar, then introduced to each colony to freely choose their phoretic hosts. After 48 hours, we sorted the three groups of bees by their colors (or in case of forages, the lack of colors) and washed them in 75% ethanol to determine the total number of mites on each group. The experiment was repeated using 4 different colonies during 2008 (East Lansing, Michigan, USA, 42.41°N, 84.28°W) and 3 colonies during 2009 (Nanchang, Jiangxi, China, 28.46°N, 115.49°E).

### Mite fecundity after feeding on different phoretic hosts

Mature daughter mites (N = 35 per group, 105 mites per trial and we conducted 4 trials in 4 colonies) were harvested from soon-to-emerge drones, so they did not have a chance to start phoretic stage yet. They were distinguished from mother mites by their lighter color. They were introduced to bees inside cages (9 × 7 × 11 cm), each with either 100 newly emerged bees (<24 hours), nurses (7 days old), or foragers (usually 21 days or older). Each cage was provided with a glass scintillation vial containing 20 ml of 50% (w/v) sucrose and another vial containing 20 ml water (both replaced daily) and incubated at 34 °C and 50% RH.

After 3 days of feeding on the different phoretic hosts, the mites were carefully recovered from each worker by using a soft brush and introduced into newly capped (within six hours) worker cells using established methods^26^ and their fecundity (number of total female offspring) or “fitness” (number of adult and deutonymph females per mother) was determined after nine days of incubation (34 °C, 50% RH). In other words, mite transfer was done at day 1 and mite fecundity was determined on day 10. The experiment was repeated using 2 different colonies during 2008 (Michigan, USA) and 2 colonies during 2009 (Nanchang, China).

In another experiment, we compared mite fecundity after they fed on hosts of different ages. Phoretic hosts were of ages of 7, 10, 14 and 20 days old at the beginning of the 3 days of mite feeding. These bees were paint-marked as newly emerged bees and then introduced into host colonies and recovered at these ages and used as phoretic hosts. Each group had 26–35 mites. This experiment was conducted in Michigan, USA.

### Data analyses

All data were presented as mean ± SE (standard error of the mean). Statistical analyses were performed using one way analysis of variance (ANOVA) by StatView (v 5.01, SAS Institute Gary, NC, USA) with significance level set at P < 0.05.

## Additional Information

**How to cite this article**: Xie, X. *et al.* Why do *Varroa* mites prefer nurse bees? *Sci. Rep.* **6**, 28228; doi: 10.1038/srep28228 (2016).
